# Fecal Microbiota Transplantation for Clostridioides Difficile Infection in Patients with Chronic Liver Disease

**DOI:** 10.1155/2020/1874570

**Published:** 2020-01-27

**Authors:** Alireza Meighani, Maryam Alimirah, Mayur Ramesh, Reena Salgia

**Affiliations:** ^1^Division of Gastroenterology and Hepatology, Henry Ford Hospital, 2799 W. Grand Blvd, Detroit, MI 48202, USA; ^2^Department of Internal Medicine, Henry Ford Hospital, 2799 W. Grand Blvd, Detroit, MI 48202, USA; ^3^Division of Infectious Diseases, Henry Ford Hospital, 2799 W. Grand Blvd, Detroit, MI 48202, USA

## Abstract

**Background:**

Fecal microbiota transplantation (FMT) is a well-established therapeutic option for patients with antibiotic resistant *Clostridioides difficile* infection (CDI). However, the efficacy of FMT in patients with chronic liver disease remains elusive.

**Aims:**

We studied the effect of FMT on chronic liver disease (CLD) patients with CDI at our tertiary medical center.

**Methods:**

A cohort of all patients who received FMT from December 2012 to May 2014 for refractory or recurrent CDI was identified. Patients were monitored for a year after FMT. Descriptive analysis was conducted to compare the effect of FMT in patients with and without CLD.

**Results:**

A total of 201 patients with CDI received FMT, 14 of which had a history of CLD. Nine of these patients exhibited cirrhosis of the liver with a mean Child-Turcotte-Pugh score of 8. CDI development in these patients was associated with recent exposure to antibiotics and was observed to be significantly different between both groups (17% of CLD patients vs. 58% in the general cohort, *p* = 0.01). Four patients with CLD received >1 FMT, of which 2 did not respond to treatment. There was no significant difference between patients with liver disease and the rest of the cohort with regard to FMT response (12/14 (87%) vs. 164/187 (88%), *p* = 0.01). Four patients with CLD received >1 FMT, of which 2 did not respond to treatment. There was no significant difference between patients with liver disease and the rest of the cohort with regard to FMT response (12/14 (87%) vs. 164/187 (88%),

**Conclusion:**

FMT is a safe and effective therapy against CDI for patients with CLD and cirrhosis.

## 1. Introduction

Antibiotic resistant *Clostridioides difficile* infection (CDI) is a major public health concern with a high death rate [1–3]. Fecal microbiota transplantation (FMT) dates back to 4^th^ century China where human fecal suspension by mouth was used to cure food poisoning and severe diarrhea [4]. FMT has been employed in patients with severe and recurrent CDI who have failed multiple attempts at conventional antibiotic therapy. Cumulative experience from case series and controlled trials shows that FMT is effective when used to treat relapsing CDI [5, 6]. The high therapeutic efficacy of FMT for recurrent CDI is an important proof of concept that substantial modification of the gut microbiota can be an effective modality for treatment of other diseases in humans such as primary sclerosing cholangitis, inflammatory bowel disease, and serious antibiotic-associated diarrhea [7–10].

Liver disease rates are steadily increasing over the years. According to the World Health Organization, about 46% of global diseases and 59% of the mortality are because of chronic diseases and almost 35 million people in the world die of chronic diseases [11]. Patients with cirrhosis and liver transplant recipients are at increased risk of developing CDI due to their frequent and prolonged hospitalizations, antibiotic and proton pump inhibitor use, multiple comorbidities, and immunosuppressant therapy [12–16]. Currently, studies on the efficacy of FMT in chronic liver disease (CLD) patients with CDI are limited. We therefore evaluated the clinical outcomes of FMT in patients with CLD at our tertiary medical center.

## 2. Methods

### 2.1. Patient Selection

Patients with recurrent or refectory CDI who received FMT from December 2012 to May 2014 were discerned and selected for analysis. The institutional review board approved the research study. Patient confidentiality was maintained prior to the analysis. Diagnosis of CLD was conferred based on the following criteria: liver cirrhosis, chronic hepatitis C infection, nonalcoholic steatohepatitis, or metabolic liver disease including Wilson's disease, alpha-1 antitrypsin deficiency, and hereditary hemochromatosis. Furthermore, patient demographics, frequency of antibiotic exposure, prevalence, and severity of CDI as well as comorbidities were incorporated during data collection. Disease recurrence and donor history were acquired from the electronic medical record database. Patients were observed for a year after FMT.

### 2.2. Patient Stratification

Diarrheal ailments are diagnosed as CDI based on a positive stool test. In our facility, CDI is determined by a two-step test. First, CDI is identified by enzyme assays that detect *Clostridioides difficile* glutamate dehydrogenase antigen as well as the *Clostridioides difficile* toxins A and B. Thus, a positive enzyme assay for *Clostridioides difficile* toxins A and B as well as the glutamate dehydrogenase antigen confers a diagnosis of CDI. Second, a discrepancy in the immunoassay results is followed by a polymerase chain reaction (PCR) to amplify the genes for *Clostridioides difficile* toxins A and B, confirming the absence or presence of CDI. Severe CDI was characterized as an elevation in serum creatinine >1.5 times above the patient's normal levels, white blood cell count ≥15,000 cells/*μ*L, or serum albumin <1.5 g/dL within 2 weeks of symptom onset [17].

CDI severity was assessed by clinical symptoms and laboratory tests. Patients without laboratory results during FMT were diagnosed with nonsevere CDI. Patients with a first episode of CDI that did not show symptomatic improvement within 5 days were defined as primary refractory CDI. Those with more than 1 episode of CDI in 1 year were considered to have recurrent CDI [18].

Oral vancomycin was administered to all of our patients before being referred for refractory or recurring CDI. Prior antibiotic use was also included in the evaluation. FMT was carried out through colonoscopy, nasogastric tube, or retention enema. FMT donors were primarily family members. However, in the absence of family donors, our facility provides a cohort of FMT universal donors to patients. All of our donors were screened for hepatitis A, B, and C; HIV and syphilis serologies; stool for bacterial PCR; ova and parasites; and *Clostridioides difficile* toxins. All of our patients received fresh FMT. Patients were considered responsive to FMT therapy when the diarrhea ceased within 7 days of treatment. Alternatively, FMT treatment was considered unsuccessful if the diarrhea failed to resolve within 7 days [19]. If the diarrhea recurred at a similar severity to the initial CDI, within 90 days of FMT, the disease was described as a relapse. Furthermore, the CDI was characterized as a new infection if the diarrhea returned 90 days after FMT treatment. Diagnosis of cirrhosis was based on imaging such as ultrasound or computed tomography of the abdomen exhibiting nodularity of the liver surface in association with an etiology for liver disease and clinical characteristics of cirrhosis. Decompensation was characterized as the presence of ascites, bleeding esophageal or gastric varices, or presence of hepatic encephalopathy.

### 2.3. Statistical Analysis

Continuous variables were shown as mean ± standard deviation while categorical variables were reported in percentages. Comparison between both study groups was conducted using two-sample *t*-tests for normally distributed numeric data. For nonnormally distributed numeric data, the Wilcoxon rank-sum tests were used. Chi-square tests were used for nonsparse categorical data. Statistical significance was denoted as *p* ≤ 0.05.

## 3. Results

Based on FMT procedure notes, 254 patients were initially identified. Patients with incomplete medical records or lack of follow-up were excluded leaving 201 patients who underwent FMT, of which 14 patients had concurrent CLD. Nine of these patients had cirrhosis with an average Child-Turcotte-Pugh score of 8. At the time of FMT, one subject had received a liver transplantation 5 months earlier. Ten of the fourteen (71%) patients with liver disease in our study were female with a mean age of 62 years. There was a significantly greater number of patients with recent antibiotic exposure and who subsequently developed CDI in the general cohort group [3/14 (21%) of CLD patients vs. 109/187 (58%) in the general cohort, *p* = 0.01] (Table 1). Response to FMT did not differ between both groups based on immunosuppression, route of FMT delivery, number of CDIs within the last 3 months, recent hospitalization, recent surgeries, or Charlson Comorbidity Index (Table 2). Severe CDI rates were similar between patients with CLD and the general cohort [5/14 (36%) vs. 45/187 (24%), *p* = 0.34]. Four patients with CLD received more than 1 FMT, of which 2 patients did not respond to treatment. Also, none of the patients with liver cirrhosis had worsening of their Child-Turcotte-Pugh score after FMT.

Finally, there was no significant difference between patients with liver disease and the rest of the cohort with regard to FMT response (12/14 (87%) vs. 164/187 (88%), *p* = 0.68).

Both of the CLD patients who did not respond to FMT had decompensated cirrhosis with a Child-Turcotte-Pugh scores of 9 and 12, respectively.

## 4. Discussion

Our study clearly shows that in patients with CLD, FMT is an effective treatment modality in treating patients with recurrent CDI. Despite the promise of FMT for treatment of recurrent CDI, current medical literature lacks robust data on the use of FMT for recurrent CDI in patients who have concurrent CLD. A study done by Allegretti et al. looked at 10 patients with primary sclerosing cholangitis, who underwent FMT to evaluate safety, liver enzymes, and microbiome profiles of primary sclerosing cholangitis patients post-FMT. The study demonstrated that FMT from a rationally selected donor is safe, increases microbial diversity, and may improve alkaline phosphatase among primary sclerosing cholangitis patients [20].

One of the fears in using FMT in patients with cirrhosis is the theoretical risk of worsening hepatic encephalopathy by increasing patients' fecal load. On the contrary, however, there have been reports that FMT has been used as a treatment option for hepatic encephalopathy. In a case reported by Kao et al., 5 FMT sessions were used to treat refractory mild hepatic encephalopathy in a patient. The theory behind its mechanism of action is an increase in microbiota diversity, specifically the beneficial taxa that are deficient in patients with recurrent hepatic encephalopathy [21]. Also, in a randomized clinical trial, FMT from a rationally selected donor reduced hospitalizations, improved cognition, and dysbiosis in cirrhotics with recurrent hepatic encephalopathy. None of the patients, however, had CDI [22]. In our cohort of patients with compensated cirrhosis, there was no worsening of disease state including hepatic encephalopathy after FMT.

Based on a multicenter retrospective study by Kelly et al. of immunocompromised CDI patients who received FMT, short-term safety data on FMT suggests that it is well tolerated among immunocompromised patients. Reasons for immunosuppression varied but included patients with inflammatory bowel disease on immunosuppressive therapy, solid organ transplant recipients, and patients undergoing chemotherapy. There were no infectious complications related to FMT in these patients [23]. In our small study, we only had 3 immunocompromised patients in the CLD group, who tolerated FMT very well with no infectious complications. Moss et al. studied long-term taxonomic and functional divergence from donor bacterial strains following FMT in immunocompromised patients. There were no complications reported after 1 year of following these patients [24]. In a study by Jalanka et al. on long-term safety of FMT, patients were followed for 3.8 years after FMT. There was no difference in the incidence of severe diseases (inflammatory bowel disease, cancer, autoimmune disease, allergy, or neurological diseases) between the patient groups [25].

The theoretical possibility of transmission of unrecognized infectious agents that cause illness years later, analogous to prior experience with hepatitis C and human immunodeficiency virus, has been raised. However, it would be assumed that such agents would induce disease in donors as well [26]. Our donors are routinely screened for a variety of infectious pathogens including HIV and agents of viral hepatitis. There is a recent case report of drug-resistant E. coli transmission from the donor, when using FMT in a clinical trial of patients with hepatic encephalopathy [27]. Therefore, based on recent FDA guidance, we have included drug resistant pathogens in our FMT donor screening. There is also theoretical risk of transmission of obesity and metabolic syndrome as previous studies have shown that transplantation of human fecal microbiota from obese subjects to rodents has been shown to transmit an obesity phenotype [28]. Also, FMT from lean subjects to obese subjects with metabolic syndrome showed an increase in insulin sensitivity [29]. Having said that, in the study done by Jalanka et al., weight gain did not differ between treatment groups [25]. We did not assess for weight change in our cohort of patients post-FMT.

Our study shows that patients with CLD were less likely to be recently exposed to antibiotics in comparison with the general population. This could potentially mean that patients with CLD are more immunosuppressed; hence, they are more at risk of acquiring CDI without being exposed to antimicrobials. Further prospective studies need to be done to elucidate this.

Our study has several drawbacks. First, this is a retrospective study where several patients were eliminated from the data analysis due to inadequate medical information. Second, treatment outcomes may have been confounded by the absence of a systematic FMT treatment regimen and through the use of multiple diverse donors. As such, individual microbiomes are diverse, comprised of multiple different microbes which are well established to vary depending on a donor's age. In our study, data on donor age was not available, thus preventing us from evaluating the effect of FMT donor age on the treatment outcome [30].

## 5. Conclusions

FMT is a safe and effective option with high rates of response in patients with chronic liver disease or cirrhosis who have CDI. This includes patients who may be organ transplant recipients and subject to immunosuppression. Recent antibiotic exposure was not a prevalent determinant for CDI in patients with liver disease. Prospective randomized controlled trials should be done, studying long-term safety and efficacy of FMT in patients with concurrent CLD and recurrent CDI.

## Figures and Tables

**Table 1:** Clinical characteristics of patients stratified by chronic liver disease status.

	Liver disease (*N* = 14)	No liver disease (*N* = 187)	*p* value
Age	61.5 (51.0-67.0)	71.0 (54.0-82.0)	0.13
Sex			
Male	4 (28.6)	72 (38.5)	0.57
Female	10 (71.4)	115 (61.5)	
Immunosuppression			
No	11/14 (78.6%)	160/187 (85.6%)	0.44
Yes	3/14 (21.4%)	27/187 (14.4%)	
Inflammatory bowel disease			
No	12/14 (85.7%)	169/187 (90.4%)	0.64
Yes	2/14 (14.3%)	18/187 (9.6%)	
Recent antibiotic exposure			
No	11/14 (78.6%)	78/187 (41.7%)	**0.01**
Yes	3/14 (21.4%)	109/187 (58.3)	
Route of fecal microbiota transplantation delivery			
Colonoscopy	5/14 (35.7%)	40/187 (21.4%)	0.76
Enema	2/14 (14.3%)	73/187 (39.0%)	
Nasogastric tube	7/14 (50.0%)	69/187 (36.9%)	
Percutaneous endoscopic gastrostomy	0/14 (0.0%)	5/187 (2.7%)	
Recent hospitalization			
No	9/14 (64.3%)	99/187 (52.9%)	0.58

**Table 2:** Response to FMT, stratified by liver disease status.

		Liver disease (*N* = 12)	No liver disease (*N* = 164)	*p* value
Age		59.5 (47.5-73.0)	72.0 (54.0-83.0)	0.17
Sex				
Male		4 (33.3)	68 (41.5)	0.76
Female		8 (66.7)	96 (58.5)	
CDI severity				
Severe		3 (25.0)	37 (22.6)	0.74
Nonsevere		9 (75.0)	127 (77.4)	
Diabetes mellitus				
No		9 (75.0)	132 (80.5)	0.71
Yes		3 (25.0)	32 (19.5)	
Hypertension				
No		8 (66.7)	87 (53.1)	0.55
Yes		4 (33.3)	77 (47.0)	
Chronic kidney disease				
No		10 (83.3)	143 (87.2)	0.66
Yes		2 (16.7)	21 (12.8)	
Cancer				
No		10 (83.3)	136 (82.9)	0.99
Yes		2 (16.7)	28 (17.1)	
Immunosuppression				
No		10 (83.3)	138 (84.2)	0.99
Yes		2 (16.7)	26 (15.9)	
Recent hospitalization				
No		9 (75.0)	92 (56.1)	0.24
Yes		3 (25.0)	72 (43.9)	
Recent surgery				
No		12 (100.0)	152 (92.7)	0.99
Yes		0 (0.0)	12 (7.3)	
Route of FMT delivery				
Colonoscopy		5 (41.7)	36 (22.0)	0.3
Enema		2 (16.7)	63 (38.4)	
Nasogastric tube		5 (41.7)	62 (37.8)	
PEG		0 (0.0)	3 (1.8)	

FMT: fecal microbiota transplantation; PEG: percutaneous endoscopic gastrostomy.

## Data Availability

The patient data used to support the findings of this study are restricted by the Henry Ford Hospital IRB in order to protect patient privacy.
